# Spatial Regulation and the Rate of Signal Transduction Activation

**DOI:** 10.1371/journal.pcbi.0020044

**Published:** 2006-05-12

**Authors:** Nizar N Batada, Larry A Shepp, David O Siegmund, Michael Levitt

**Affiliations:** 1 Program in Biophysics, Stanford University, Stanford, California, United States of America; 2 Department of Statistics, Rutgers University, Piscataway, New Jersey, United States of America; 3 Department of Statistics, Stanford University, Stanford, California, United States of America; 4 Department of Structural Biology, Stanford School of Medicine, Stanford, California, United States of America; European Molecular Biology Laboratory, Germany

## Abstract

Of the many important signaling events that take place on the surface of a mammalian cell, activation of signal transduction pathways via interactions of cell surface receptors is one of the most important. Evidence suggests that cell surface proteins are not as freely diffusible as implied by the classic fluid mosaic model and that their confinement to membrane domains is regulated. It is unknown whether these dynamic localization mechanisms function to enhance signal transduction activation rate or to minimize cross talk among pathways that share common intermediates. To determine which of these two possibilities is more likely, we derive an explicit equation for the rate at which cell surface membrane proteins interact based on a Brownian motion model in the presence of endocytosis and exocytosis. We find that in the absence of any diffusion constraints, cell surface protein interaction rate is extremely high relative to cytoplasmic protein interaction rate even in a large mammalian cell with a receptor abundance of a mere two hundred molecules. Since a larger number of downstream signaling events needs to take place, each occurring at a much slower rate than the initial activation via association of cell surface proteins, we conclude that the role of co-localization is most likely that of cross-talk reduction rather than coupling efficiency enhancement.

## Synopsis

Cells coordinate activities with their neighboring cells by releasing and responding to chemical signals, such as hormones and growth factors. These extracellular signals often are transmitted to the intracellular regulatory machinery through associations of freely diffusing cell surface receptors whose random movement (Brownian motion) results from collisions with thermally agitated lipid molecules. Receptors on the cell surface signaling proteins are co-localized transiently on membrane patches, but how such spatial restriction contributes to signal transduction is unclear. Batada and colleagues set out to determine which is more likely to be the biophysical function of co-localization: increasing coupling efficiency of slowly diffusing membrane proteins or reducing cross talk along pathways with common intermediates. Using a mathematical model to describe Brownian motion–based association of receptors on a 2-D spherical surface, the authors show that the rate of signal transduction initiation is extremely fast compared with the cytoplasmic signal relay events, even when the number of receptors is low. Because enhancement of a process that is already fast is not beneficial to the cell, minimization of detrimental cross talk among pathways is the most likely reason why cells need to co-localize plasma membrane signaling proteins.

## Introduction

The surface of a eukaryotic cell is embedded with a diversity of receptors that serve as the primary conduits for transmission of environmental information into the cell's signaling network. Binding of extracellular chemical signals to receptors and the subsequent interaction of these ligand occupied receptors constitute primary and necessary events in the activation of many signal transduction pathways[1–3]. For example, in mammalian cells, a common mode of activation of intracellular pathways in response to extracellular peptide hormones is through dimerization of ligand-occupied receptor monomers such as transforming growth factor receptors receptors, epidermal growth factor receptors, and members of the receptor tyrosine kinase family [4].

Why has a dimerization mechanism evolved when a single monomer could do just as well? Dimerization may aid signaling via presentation of more exposed intracellular signaling domains, creating larger and more diverse interfaces for recognition of cytosolic transducer and linker molecules [5]. Another advantage may include the ability to generate diverse combinatorial responses to different inputs [6]. However, the need for an additional binding partner can result in signaling response delays due to slow diffusion of proteins in the lipid bilayer. Moreover, reuse of membrane proteins in different pathways can result in nonspecific activation or cross talk.

How does the cell ensure reliable and fast response for the dimerization triggered signal transduction initiation? The picture of a cell surface membrane as a sea of lipids in which transmembrane proteins diffuse freely as suggested by the fluid mosaic model [7] is too simple [8–10]. Static structural proteins such as cytoskeleton fences and obstacles embedded in the lipid bilayer hinder lateral mobility of transmembrane proteins causing anomalous diffusion [11,12]. In addition, cells can transiently confine membrane proteins to localized regions within the lipid bilayer via specialized high viscosity membrane patches composed of cholesterol and sphingolipids known as lipid rafts [13] and caveolae [14–16]. The fact that cell surface membrane receptors and signaling molecules are enriched in these membrane patches [17,18] and that disruption of these structures lead to abnormal signaling and disease [19] suggests a central cell-signaling role for these domains.

How does co-localization of cell surface proteins affect signal transduction? In the context of signal transduction, there are potentially two biophysical reasons for limiting the range of membrane proteins: first, to insulate pathways that share common intermediates (i.e., to increase the specificity of response), and second, to enhance the activation rate by increasing coupling efficiency of the membrane embedded components of these pathways. The enhancement of interactions may be important because the diffusion coefficient of receptors and proteins on the cell surface membrane are generally more than two orders of magnitude smaller than in the cytoplasm [20,21]; the resulting slow rate of association may prevent a cell from responding to rapidly varying extracellular signals. To determine which of these two hypotheses is more likely, we created a mathematical model of diffusion-based encounters of cell surface proteins. We reason that if in the absence of diffusion barriers the rate of association of membrane proteins that are present in only few copies is not a rate-limiting step in the overall signal transduction process, then it is unlikely that the role of co-localization is to enhance interaction rates as there is no benefit in enhancing the rate of an event that is not rate-limiting.

## Results

### Brownian Motion of Proteins on a Spherical Surface

How do proteins diffuse on the cell surface? Erratic motions of macromolecules are caused by collision with thermally powered molecules in the medium; in the case of cell surface receptors, movement is driven by incessant collisions with the lipid molecules of the membrane bilayer. Evidence for the existence of obstacles to protein movement has been obtained for several cell types by imaging experiments [22,23]. Simulations of diffusion in the presence of obstacles and nonspecific binding have been done to study how anomalous diffusion arises [11,24]. As it is not clear whether obstacles are present in all cells or only for some specialized cells, we make a commonly made assumption that the diffusion is normal, i.e., as described by the fluid mosaic model [7,25] but expect that the results derived here will be valid when the cell surface is not overly crowded. To model diffusion in a crowded environment requires explicit information regarding spatial non-uniformity, making analytical treatment of anomalous diffusion intractable.

**Figure 1:**
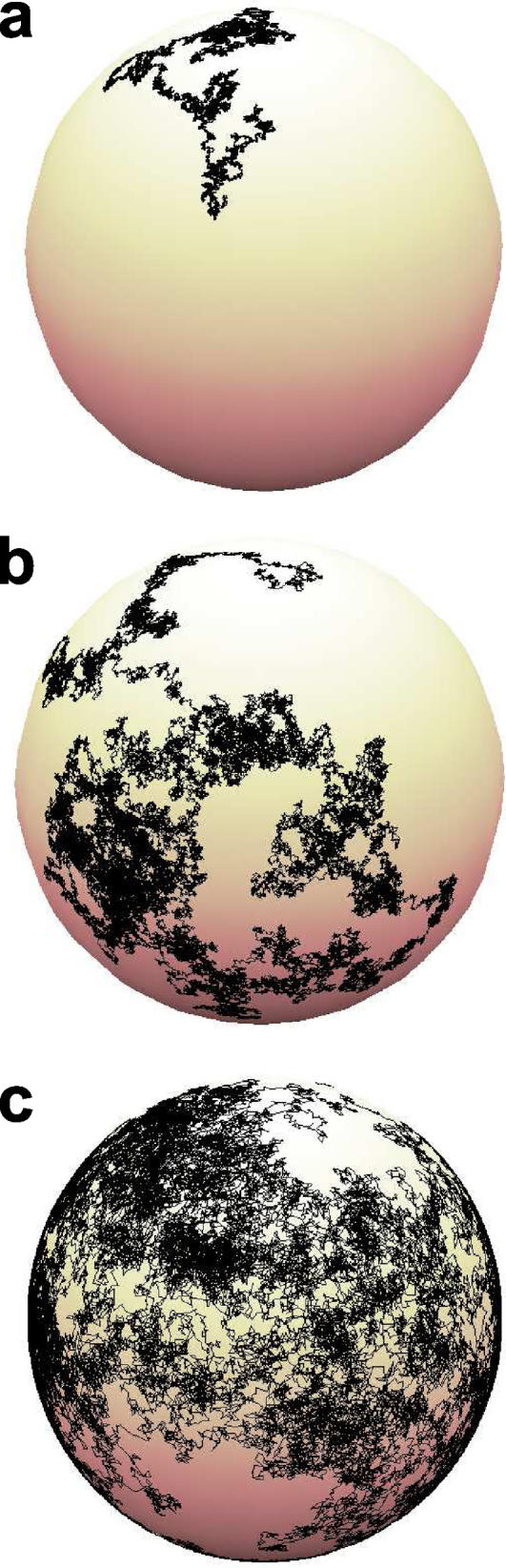
Brownian Motion on the Surface of a Spherical Cell as Given by Equation 1 Figure 1 verifies that the SDE constructed in Equation 1 correctly describes the random diffusive movement of membrane bound particles. Shown are trajectories of a diffusing particle on the surface of a cell with 10 *μm* radius for a fixed time with different diffusion coefficients. (A) *D* = 0*.*03 *μm* ^2^/*s,* (B) *D* = 0*.*3 *μm* ^2^/*s,* (C) *D* = 3 *μm* ^2^/*s*.

Suppose (*X*(t),*Y*(t),*Z*(t)) is a Brownian diffusion on a sphere of radius *R* with a variance of *σ* ^2^ *dt* = (*dX*)^2^ + (*dY*)^2^ + (*dZ*)^2^ in *dt* seconds. Our construction of the following stochastic differential equation (SDE)

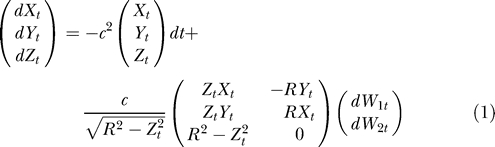
describes the time-evolution of the position of a randomly diffusing particle on the surface of a sphere with *c* = *σ*/*R* 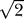 . In Equation 1 , *W_i_*(*t*) is a one-dimensional standard Brownian motion. Using Ito calculus, we verified that 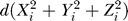 ≡ 0, so that the motion of a particle initially on a sphere (i.e., *X_i_* (0)^2^ + *Y_i_* (0)^2^ + *Z_i_* (0)_2_ = *R* ^2^) remains on the same sphere for all time. The corresponding SDE for the angles has been studied extensively in the probability [26] and physics community [27], but we need the SDE in Cartesian coordinates given above as it facilitates the analytical derivation of the mean interaction rate in the presence of endocytosis (or “death”) and exocytosis (or “birth”). We verified the correctness of Equation 1 and its first two moments by simulation (Figure 1).

### Signal Transduction Activation via Receptor Dimerization

We use the term dimerization to describe the physical association event between freely diffusing cell surface proteins and receptors, and limit the scope of our model to dynamic hetero-dimerization—those situations where protein–protein interaction event itself is part of a decision point in the signaling process. We make the following two assumptions: 1) signal transduction activation via interaction of membrane proteins is diffusion controlled; 2) exocytosis (the process that inserts proteins into the cell surface) and endocytosis (the process that removes proteins from the cell surface) activities are constitutive (i.e., occurring continuously and independently of the presence of ligand) and uniform throughout the cell surface [28,29].

**Figure 2:**
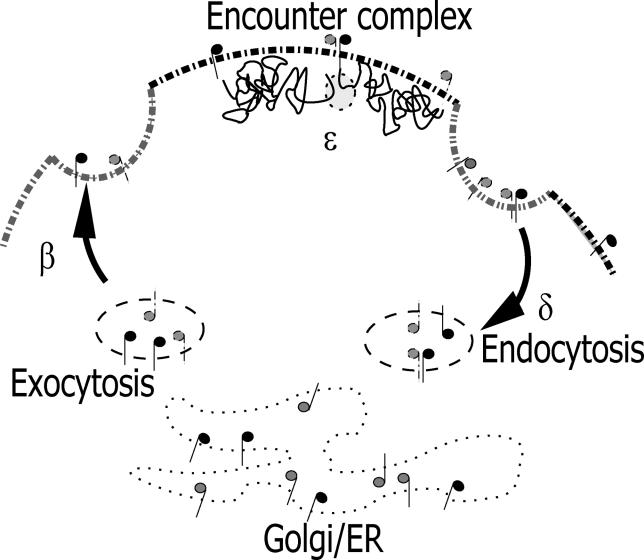
Illustration of the Model for the Receptor Dimerization Process As soon as receptors are introduced onto the cell surface, they undergo isotropic Brownian motion. At any given time, there is a small constant probability that a protein may be removed from the cell surface via endocytosis. If at any time two receptors of different type come within close proximity, they may form an active dimeric complex with probability *p*.

The term *diffusion controlled* in assumption 1) means that the overall signal transduction activation kinetics are controlled by the transport mechanism that brings the reactive pairs together. In other words, *k_on_* for the reaction rate is determined only by the diffusion dependent encounter rate of the reactants. Although there are some polarized cells, such as epithelial cells, for which there is spatial preference and a nonconstant rate of exocytosis [30], it is not possible to take this into account as the rates of these processes depend on complex cytoplasmic events that are difficult to specify. Assumption 2 also implies that endocytosis and exocytosis do not depend on each other. We note that membrane proteins undergo rotational diffusion concurrently with translational diffusion, and once a pair of proteins has interacted in either a proper or an improper orientation, they may not form an active complex; however, they are able to repeatedly interact with each other in quick succession. If a compatible orientation of these pairs is obtained within this time interval, they may form an active complex or else they escape from each other. In our model, we do take into account that only a small fraction of all encounters may lead to active complexes. Figure 2 illustrates the model of receptor dimerization that we use. Our goal is to determine if the active complex formation rate is slow or not so that we can decide whether co-localization can potentially help improve the coupling efficiency of interacting partners.

### Encounter Rate of Proteins on the Cell Surface

Using the receptor dimerization model described in the previous section, we would like to derive the mean rate at which different types of receptors interact at steady state upon uniform activation by some extracellular chemical signal. Simulation studies [31–33] have been done to understand the dimerization of cell surface receptors; these studies modeled the surface geometry as a planar surface with infinite boundary and did not consider the role of endocytosis and exocytosis. An advantage of an analytical model over a simulation model is that the former explicitly defines the contribution of parameters toward modulating the dimerization rate, and as a result can be applied much more generally. We first derive the rate at which two uniformly randomly placed freely diffusing proteins subject to removal via constitutive endocytosis meet on a spherical cell surface, and then generalize this result in the case when there are steady state levels of proteins of each type.

For a pair of Brownian motions (*X_i_*(*t*),*Y_i_*(*t*),*Z_i_*(*t*)), *i* = 1,2 with different diffusion coefficients *σ_i_,* consider the latitude of the second relative to the first in a coordinate system in which the north pole is considered always to be at the location of the first protein. This is the same as looking at the inner product or the angle between two Brownian motions on a sphere. Due to the spherical symmetry, the third coordinate, *Z*(*t*), of Equation 1 describes the time-evolution of distance between these two proteins (see Protocol S1).

If *ɛ* is the sum of radii of two interacting particles, then let 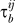 be the first encounter time between a particle *i* of type I and a particle *j* of type II with *b* = 1 – *ɛ*. Formally, 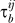 = inf{*t* ∈ *Z_ij_*(*t*) = 1 − ɛ}, is the first time when a Brownian starting uniformly with unit diffusion constant hits the *ɛ* cap of the north pole of a unit sphere. Note that if *Z*(0) = *z* is the starting position of the distance process, then the initial distance between a particle *i* of type I and a particle *j* of type II is 1 – *z*.

Let *δ_i_* be the death rate of particle *i* and let *δ* = *δ*_1_ + *δ*_2_. If 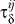 denotes the death time of particle *i* or particle *j,* whichever dies sooner, then Pr(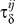 ∈ *dt*) = *δe* ^−*δt*^ *dt* because the minimum of two independent exponentially distributed random variables is also exponentially distributed. The probability that there will ever be an interaction between a given pair of proteins is the same as the probability that the *ɛ*-meeting time of these particles is less than the death time of either of them. Formally we are interested in computing 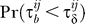 , the probability that there is an encounter between proteins *i* and *j*. Upon conditioning on 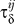 , and using the independence of degradation and movement, we get

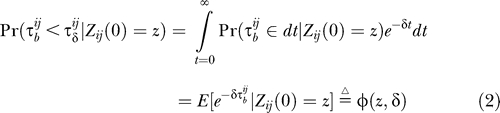
where *E*[·] is the expectation operator. Note that because the degradation process is memoryless, the interaction probability is simply the Laplace transform of the first interaction time, *φ*(*z, δ*).

After accounting for steady state levels of proteins of each type, rescaling to general radius *R* and diffusion parameter *D,* the expected rate of interaction of freely diffusing cell surface proteins of different types, *m,* is given by (derivation in Materials and Methods):

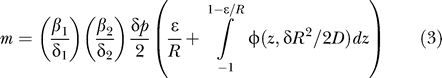
where *D* is the sum of the two diffusion coefficients and *p* is the probability that an encounter results in an active complex, i.e., with mutually compatible orientation and sufficient energy to overcome the activation barrier required for binding reaction to take place. The remaining integral is not easy to give in a closed form as it involves ratios of Legendre functions, but is solvable via numerical integration (a matlab program to compute the interaction rate is given in Protocol S2). As required, the rate conforms to reasonable values in limiting cases: as *δ* → ∞, *m* → 0, and as *δ* → 0 *m* simplifies to Equation 4 given below. Note, however, that the *φ* function depends on parameters in addition to *δ* such that increase in *R* or reduction in *σ* can compensate for reduction in *δ.*

For certain combinations of physiologically relevant parameters, *φ'*s second argument, *δR* ^2^/2D, is small (i.e., ≤0.1). In this special case, Equation 3 can be approximated with a simpler equation. As *λ = δR* ^2^ /*σ* ^2^ → 0, we see from the approximation exp (−*λτ) =* 1 − *λτ* + O(*λ* ^2^) that the product 
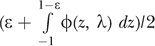
 is of the form 1 − *Cλ* + O(*λ* ^2^) where *C* is evaluated as an integral of *E_z_*(*τ*_1−*ɛ*_) *=* 2log((1 − *z*)/*ɛ*) with respect to *z*. Replacing *ɛ* with *ɛ*/*R* to scale back to the sphere of radius *R* and integrating to get C = *ɛ*/*R +* 2log(2*R*/*ɛ*) – 2 reduces Equation 3 to

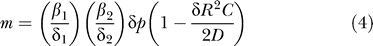
Note that the above approximation holds only if *δ* is much less than 2*D*/*R* ^2^ *C*.

Since the number of proteins of type *i* at steady state has a Poisson distribution with mean *β_i_*/*δ_i_,* the forward rate constant (with units of *s* ^−1^ per complex) is given by

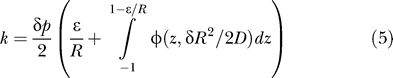
or *k* = *δp*(1 − *δR* ^2^C/2*D*) for the special case mentioned.

For a mammalian cell, *D* is in the range 0.5 − 0.05 *μm* ^2^ *s* ^−1^ [34–36], *R* is in the range 2 − 10 *μm* and the endocytosis rate varies from 10^−4^ − 10^−2^ *s* ^−1^ [28,37]. All the parameters needed to compute the rate of active complex formation using Equation 3 have been measured experimentally except *p;* however, we can make a rough estimate for *p* using geometric and biophysical arguments. For example, if one quarter of the exposed surface area of a transmembrane protein contains the interaction site, then on average one sixteenth of the encounters occur in proper orientation, as rotational diffusion is random. Short-range and long-range forces such as van der Waals, hydrophobic, and electrostatic forces can serve as sources of adhesion interactions thereby guiding and prolonging the duration of collisions, giving the potential partners many opportunities to seek out correct mutual orientations for binding [38]. According to such arguments we can reasonably assume that on average one in ten interactions lead to an active complex, i.e., *p* ≅ 0.1. Note that the fraction of trajectories that lead to an active complex formation is expected to be much larger on a cell surface then in the cytoplasm due to the reduced degree of freedom, slow diffusion rate, and orientation constraints imposed on transmembrane proteins by the 2-D surface of the lipid bilayer. Using the physiologically relevant parameters for a typical mammalian cell, i.e., *R* = 10 *μm, ɛ* = 0.01 *μm, D* = 0.1 *μm* ^2^ *s* ^−1^ and a half-life of 30 min, only 160 receptors of each type are required to have an active complex formation rate of 1 S^−1^. As the actual, physiological abundance levels of these receptors are much higher, this means that a mammalian cell can theoretically respond to a uniformly random stimulus lasting only a few seconds. Thus, the first step in a dimerization-based signal transduction takes place on a relatively large cell almost instantaneously even with low receptor abundance.

### Comparison of the Signaling Time on the Cell Surface and in the Cytoplasm

Is the activation of a signal transduction pathway via dimerization of receptors on the cell membrane the rate-limiting step in the overall signaling process? The standard chemical kinetics measure of association, given in units of *M* ^−1^ *s* ^−1^, requires a volume element, making it difficult to compare the interaction rate on a 2-D surface with the interaction rate in the cytoplasm. Degradation poses another complication: the expected interaction time in the presence of degradation can be infinite as there is a non-zero probability that two particles can never meet which occurs when one of a pair is degraded prior to meeting. We therefore compare the expected time, assuming that the degradation rate is extremely small. In such cases, the mean expected interaction time of uniformly distributed proteins on a spherical cell surface is *R* ^2^(log(*R*/*ɛ*) + *γ*)/*D*_(2)_ (see Materials and Methods) and *R* ^3^/3*ɛD*_(3)_ in the cytoplasm. In these equations, *D*_(2)_ is the diffusion coefficient on the cell surface and *D*_(3)_ is the diffusion coefficient in the cytoplasm. Using *R* = 10 *μm, D*_(2)_ = 0.1 *μm* ^2^ *s* ^−1^, *D*_(3)_ = 5 *μm* ^2^ *s* ^−1^, and *ɛ* = 0.01 *μm,* the time required for two proteins to interact on the cell surface is two-thirds of the time required for meeting of two proteins in the cytoplasm [39], assuming no spatial restrictions and diffusion-controlled reactions.

A typical signal transduction involves multiple distributive interactions, such as recruitment of signaling proteins to activated receptor complexes and the subsequent protein–protein interactions and post-translational modifications before the activated transcription factors diffuses into the nucleus and bind to an appropriate gene promoter. As the latter step is relatively quick [40,41] (assuming target gene is accessible), the short delay for cell surface receptor association implies that the overall transcriptional response time to an extracellular stimulus is mainly determined by the time required by cytoplasmic proteins to relay the activation signal from the cytoplasmic domain of active receptor complexes to the nuclear pore. In light of these considerations, one would expect that signaling pathways for cellular response to (unpredictable) extracellular stress which require quick response likely have fewer cytoplasmic protein–protein interactions than pathways of cellular processes that respond to general (programmed) developmental cues such as growth factors and hormones.

## Discussion

Inspired by the recent experimental findings that maintenance of dynamic spatial inhomogeneity of membrane proteins is achieved by transient confinement in high viscosity membrane patches such as lipid rafts [13] and caveolae [14,42], we set out to determine which of the two possible alternatives, increasing response speed or achieving pathway insulation, is the more likely biophysical role of these diffusion restricting structures. Given that diffusion coefficients of membrane receptors are hundreds of times smaller than the diffusion coefficient of similarly sized proteins in the cytoplasm, we initially hypothesized that membrane domains function to increase response rate by enhancing the coupling efficiency of slowly diffusing membrane proteins. However, we rejected this hypothesis after finding that the cell surface protein interaction rate is extremely high even when proteins are on a relatively large cell and present in only a few hundred copies. Moreover, despite a much smaller diffusion coefficient, the mean interaction delay for association of two proteins on the cell surface is much smaller than the interaction delay for association of two proteins in the cytoplasm [39], contrary to a previous suggestion [5].

How sensitive is our conclusion to variations in parameters? A key parameter in Equation 3 is *p,* the probability that two receptors that have encountered will form an active complex; however, no experimental estimate is available for it. One way to account for variations in the active complex formation efficiency is to reduce the interaction distance, *ɛ*. However, as the expected interaction time depends on log(*ɛ*), the change in the mean interaction time should be relatively robust to modest changes in *ɛ*. Diffusion coefficients can also vary by an order of magnitude; however, we used a reasonably low value, so our conclusion is conservative with respect to this parameter.

We note some limitations of our model. First, we have assumed constitutive endocytosis. But in some cases endocytosis is regulated such that activated receptor complexes are preferentially internalized [43]. Second, we have assumed that signal transduction activation is diffusion limited. When the activation energy for the active complex formation is high, the rate given in Equation 3 represents the fastest possible activation rate achievable. Finally, as mentioned previously, we have assumed that proteins undergo isotropic diffusion, but it is known that some membrane proteins undergo anomalous diffusion for some cell types [11,44,45] and even “hop” from one membrane domain to another [22]. We leave these refinements for future work.

Although high viscosity patches such as lipid rafts and caveolins are shown to play a key part in membrane trafficking and signal transduction, their functional role is unclear. Our finding that dimerization-based signal transduction activation is not the rate-limiting event in the overall signal transduction in response to extracellular signals, provides theoretical support against the view that membrane domains function to enhance coupling efficiency of receptors. We propose that the more likely function of plasma membrane co-localization mechanisms such as receptor clustering is to minimize nonspecific cross talk between disparate pathways that share membrane components.

## Materials and Methods

### Derivation of the interaction rate.

In this section, we give a detailed derivation of the interaction rate given in Equation 3.

First note that if (*x*(*t*), *y*(*t*), *z*(*t*)) is a standard dimensionless Brownian motion on the unit sphere with unit diffusion constant, then the rescaling for arbitrary variance coefficient *c* ^2^ and radius *R* is given by

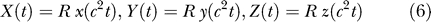
For a pair of Brownian motions (*X_i_*(t),*Y_i_*(t),*Z_i_*(t)) with different diffusion constants, *σ_i_,* the latitude of the second relative to the first in a coordinate system in which the north pole is considered always to be at the location of the first particle is precisely the process *Z*(*t*) above with *c* ^2^ = 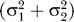 /2*R* ^2^. Thus, the Ito equation for the distance between particle *i* of type I and particle *j* of type II (or equivalently the sin *θ_ij_*(*t*) where *θ_ij_*(*t*) is the elevation angle between the particles) is given by

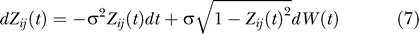
with *σ* ^2^ = 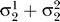 . Now, *Z*(*t*) comes within 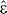 = *ɛR* of the north pole on the *R* sphere if and only if *z*(*c* ^2^ *t*) comes within *ɛ* of the north pole of the unit sphere, and this happens at dimensionless time *τ*_1−*ɛ*_/c^2^.

Let 
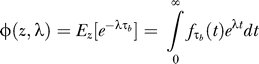
 be the Laplace transform of *τ_b_,* the time of *z*(*t*) to hit *b* = 1 − *ɛ* starting from *z*. By standard arguments, the problem reduces to the following boundary value problem

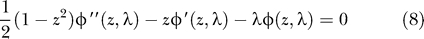
with boundary conditions *φ*(1 − *ɛ*, *λ*) = 1 and *φ* bounded as *z* → −1 (note that although there are singularities at 1 and −1, these points are never attained). Solutions of Equation 8 are Legendre functions [46]. If *f*(*z*) is a solution of Equation 8, then *Y*(t) = *e* ^−*λt*^ *f*(Z(*t*)) is a Martingale [47]. Since there are singularities at 1 and −1, where *f*(*z*) is unbounded, it may not be correct to say that *Y*(*t*) is a Martingale. However, *Y* (min(*τ*,*t*)) is a bounded Martingale (by Ito's lemma) and this is sufficient for our purpose. If *f*(*z*) is the solution, then so are the even and the odd functions *f*(*z*) + *f*(−*z*) and *f*(*z*) − *f*(−*z*). Let *f*(*z*) denote the even solution and let *g*(*z*) denote the odd solution. Up to a multiplicative constant, we have

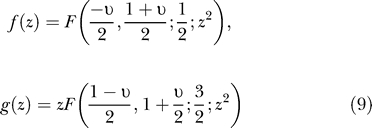
where *F*(*a*, *b*; *c*; *z*) is the hypergeometric function and *λ* = *υ*(1 *+ υ*)/2, with either root *υ* [46].

To express *φ* in terms of *f* and *g,* let *b* = 1 − *ɛ* (note that in this notation *τ*_1−*ɛ*_ ≡ *τ_b_*) and let *a* be a number which will eventually tend to −1. For *a* < *z* < *b* and *τ* = min(*τ*_a_, *τ*_b_), the Optional Sampling Identity [47] for *τ* gives

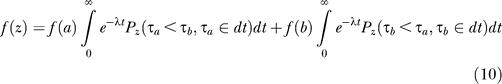
and a similar equation for *g*(*z*). The solution to these two equations with two unknowns is


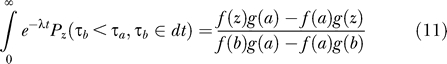


Using the formula 15.3.10 in Abramowitz and Stegun [46] on page 559, we can approximate the hypergeometric function as

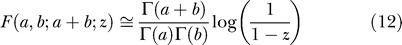
as *z* ↑ 1. Using this approximation, we get

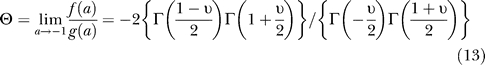
giving an expression for the desired Laplace transform

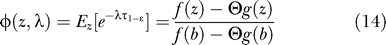
for *z* < *b* = 1 − *ɛ.*

Finally, the rate at which particles of different types with birth rates *β_i_,* death rates *δ_i_*, and diffusion rates *σ_i_* meet on a sphere of radius *R,* is given by (noting that we need to consider both the cases where either one of them is born first)

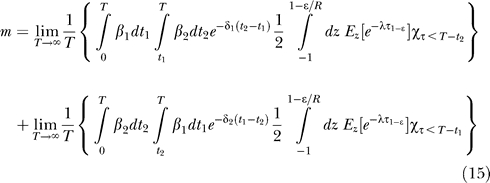
where *χ* is the indicator function and *λ = R* ^2^ *δ/σ*. Integration of this equation twice, gives the desired Equation 3.

### Mean interaction time.

In this section, we derive the mean interaction time of two cell surface proteins starting *z* apart in latitude, assuming that there is no endocytosis (i.e., death) or exocytosis (i.e., birth). Let *τ*_1−*ɛ*_ be the first time proteins interact on a unit sphere. Then let *f*(*z*) = *E*[τ_1−*ɛ*_|*Z*(0) = *z*]. After Taylor expansion and using the fact that *E*[*dW_t_*] = 0, *E*[*dW_t_dt*] = 0, *E*[(*dW_t_*)^2^], we find that *f*(*z*) must satisfy (1 – *z* ^2^)*f"*(*z*)/2 − *zf′*(*z*) + 1 = 0 with boundary conditions *f*(1 − *ɛ*) = 0 and *f′*(−1). The solution of this differential equation is *f*(*z*) = 2log((1 − *z*)/*ɛ*).

If the distance between two proteins on a sphere is uniformly distributed, then the expected value of the meeting time (after rescaling with *τ_R_*_(1−*ɛ*)_ = *R* ^2^ *τ*_1−*ɛ*_/*σ* ^2^) is given by

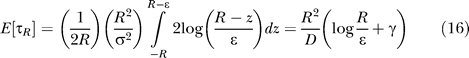
where *γ* = log2 − 1 + *ɛ*/*R* is negative and cannot be ignored as the term *γR* ^2^ */D* is not negligible for physiologically relevant parameters. This solution is similar to a previously derived result, *E*[*τ*] = (*R* ^2^/*D*)log(*R*/*ɛ*) [48], which assumed a disc-shaped region with a sink in the center and a constant boundary condition at *R*.

## Supporting Information

Protocol S1 Ito Equation Satisfied by the Interaction Process (67 KB DOC)

Protocol S2 A Program to Compute the Receptor Dimerization Rate Given in Equation 3
A matlab program to compute the rate at which receptors/proteins interact (on a spherical cell surface).
(38 KB DOC)

## Abbreviations

SDE: stochastic differential equation
