# Supplementary material for: Spatial Regulation and the Rate of Signal Transduction Activation
Source: PLoS Comput Biol. 2006 May 12;2(5):e44. doi: 10.1371/journal.pcbi.0020044 (PMC1458967; doi:10.1371/journal.pcbi.0020044)
Supplement: Supplementary file 1 [file pcbi.0020044.sd001.doc]

Supplementary Information

Batada et al 2005 *PLoS Computational Biology*

Spatial regulation of signal transduction activation

Protocol S1.

Ito equation satisfied by the interaction process.

As seen earlier, the Ito process defined by Equation 1 describes the Brownian motion of an individual protein. For clarity, we have suppressed the dependence on time, i.e., we write *X* instead of *X*(*t*). We now show that the projection of one Brownian onto another is *U_t_* = *X*_1_*_t_X*_2_*_t_* + *Y*_1_*_t_Y*_2_*_t_* + *Z*_1_*_t_Z*_2_*_t_* and satisfies the Ito equation $dU_{t}=-\sigma^{2}U_{t}dt+\sigma\sqrt{1-U_{t}^{2}}dW_{t}$ with $\sigma=\sqrt{\sigma_{1}^{2}+\sigma_{2}^{2}}$. This Ito equation is a 1D-derived Brownian motion that describes the interaction process of two independent spherical Brownians.

Since all four Brownians, *W_ij_* for *i* = 1,2 and *j* = 1,2, are independent,

$dU=X_{1}dX_{2}+X_{2}dX_{1}+Y_{1}dY_{2}+Y_{2}dY_{1}+Z_{1}dZ_{2}+Z_{2}dZ_{1}$ (17)

Substituting equations for each component gives $\begin{matrix} dU=-\sigma^{2}Udt+\frac{\sigma_{2}}{\sqrt{1-Z_{2}^{2}}}\left(Z_{2}X_{1}X_{2}+Z_{2}Y_{1}Y_{2}-Z_{1}(1-Z_{2}^{2})\right)dW_{1,2}+ \\ \frac{\sigma_{1}}{\sqrt{1-Z_{1}^{2}}}\left(Z_{1}X_{1}X_{2}+Z_{1}Y_{1}Y_{2}-Z_{2}(1-Z_{1}^{2})\right)dW_{2,1}+\frac{\sigma_{2}}{\sqrt{1-Z_{2}^{2}}}\left(-X_{1}Y_{2}+X_{2}Y_{1}\right)dW_{2,2}+\frac{\sigma_{1}}{\sqrt{1-Z_{1}^{2}}}\left(-X_{2}Y_{1}+X_{1}Y_{2}\right)dW_{1,1} \end{matrix}$(18)

Using the rule $aW_{1t}+bW_{2t}=\sqrt{a^{2}+b^{2}}W_{3t}$ and the definition of *dU_t_* reduces the above equation to

$dU=-\sigma^{2}Udt+\sqrt{\frac{\sigma_{2}^{2}}{1-Z_{2}^{2}}}\left[\left(Z_{2}U-Z_{1}\right)^{2}+\left(X_{1}Y_{2}-X_{2}Y_{1}\right)^{2}\right]+\frac{\sigma_{1}^{2}}{1-Z_{1}^{2}}\left[\left(Z_{1}U-Z_{2}\right)^{2}+\left(X_{1}Y_{2}-X_{2}Y_{1}\right)^{2}\right]$ (19)

If $\left(Z_{2}U-Z_{1}\right)^{2}+\left(X_{1}Y_{2}-X_{2}Y_{1}\right)^{2}=\left(1-Z_{2}^{2}\right)\left(1-U^{2}\right)$, and since $X_{j}^{2}+Y_{j}^{2}+Z_{j}^{2}=1$ and $U=X_{1}X_{2}+Y_{1}Y+Z_{1}Z_{2}$, this means that $(X_{1}X_{2}+Y_{1}Y_{2})^{2}-(X_{1}^{2}+Y_{1}^{2})(X_{2}^{2}+Y_{2}^{2})+(X_{1}Y_{2}-X_{2}Y_{1})^{2}=0$, which is identity, proving our claim that projection of one spherical Brownian onto another is just the *Z_t_* process.
