# Supplementary material for: Spatial Regulation and the Rate of Signal Transduction Activation
Source: PLoS Comput Biol. 2006 May 12;2(5):e44. doi: 10.1371/journal.pcbi.0020044 (PMC1458967; doi:10.1371/journal.pcbi.0020044)
Supplement: Supplementary file 2 [file pcbi.0020044.sd002.doc]

```
function mean_rate=encounter_rate_sphere(N1,N2,D1,D2, R)
%
% Note: rename the file from "Protocol S2" to "encounter_rate_sphere.m"
%
% A matlab program to compute the rate at which receptors/proteins interact
% on a (spherical cell surface)
%
%
% For details see:
%
% Batada et al (2005)
% Spatial Regulation of Signal Transduction Activation
% PLoS Computational Biology
%
% PARAMETERS: (notation: micro = u , seconds = s)
%    N1,N2 = number of cell-surface receptor monomers/proteins of type 1 (example: 100)
%    D1,D2 = diffusion coefficient of receptor monomers (units of um^2/s) (example: 0.1)
%    R = cell radius in um
% OUTPUT:
%    mean rate at which cell-surface receptors interact in units of
%      complexes or dimers per second
%
% Report problems to Nizar at nizar.batada@utoronto.ca


%%%%%%%%%%%%%%%%%%%%%%%%%%%%%%%%%%%%%%%%%%%%%%%%%%
% USER DEFINED PARAMETERS
%%%%%%%%%%%%%%%%%%%%%%%%%%%%%%%%%%%%%%%%%%%%%%%
halflife1 = 30;                          % of receptor type 1 in minutes (endocytosis)
halflife2 = 30;                          % of receptor type 2 in minutes (endocytosis)

epsilon = 0.01;                         % interaction distance (units of um)

alpha = 0.1;                            % probability that an interaction
                                        % results in an active complex
                                        % (see manuscript for how this is computed)


%%%%%%%%%%%%%%%%%%%%%%%%%%%%%%%%%%%%%%%%%%%%%%%%%%%%%%%%%%%%%
% DON'T CHANGE ANYTHING BELOW UNLESS YOU KNOW WHAT YOU ARE DOING
%%%%%%%%%%%%%%%%%%%%%%%%%%%%%%%%%%%%%%%%%%%%%%%%%%%%%%%%%

delta1 =  log(2)/(60*halflife1);         % "death" rate (units of 1/s)
delta2 =  log(2)/(60*halflife2);

beta1 = delta1*N1;                      % "birth" rate (recall that at steady state, average number of
                                        % receptors is given by beta/delta (see manuscript)
beta2 = delta2*N2;

sigma1 = sqrt( 2*D1);                   % Diffusion parameter
sigma2 = sqrt( 2*D2);
sigma = sqrt(sigma1^2 + sigma2^2 );

b = 1-epsilon;
lambda = (delta1+delta2)*R^2/sigma^2;
nu = (-1 + sqrt(1 - 8*lambda))/2;


theta = -2*(gamma( (1-nu)/2 ) * gamma( 1 + nu/2) ) / ( gamma( - nu/2 )*gamma((1+nu)/2));

fb = hypergeom([-nu/2 , (1+nu)/2],[1/2], b^2);
gb =  b * hypergeom([(1-nu)/2, 1+nu/2],[3/2],b^2);

ET = quadl(@phi_integrand,-0.999999,1-epsilon/R,[],[],nu,theta,fb,gb);

mean_rate = alpha*beta1*beta2*(1/delta1 + 1/delta2)*(1/2)*(epsilon/R + ET);


%------------------------------------------
function out=phi_integrand(t,nu,theta,fb,gb)
fz=hypergeom([-nu/2, (1+nu)/2],[1/2],t.^2);
gz=t.*hypergeom([(1-nu)/2,1+nu/2],[3/2],t.^2);
out= ( fz - theta* gz )./ ( fb - theta.*gb);
```
